# Hospital and Institutional News

**Published:** 1916-11-25

**Authors:** 


### HOSPITAL AND INSTITUTIONAL NEWS.

### DINNER TO MR. CLIVE BRIDGEMAN, M.P.

The complimentary dinner (mentioned on p. 151 of our last issue) given by the Hospital Officers' Association to Mr. W. Clive Bridgeman was a very pleasant occasion, and attracted a gathering who no doubt were rather agreeably surprised when the speeches did not deal exclusively with the relief to hospitals in respect of the duty on rectified spirits. That subject naturally received first attention, and the guest of the evening gave a short history of a movement which, overcoming the curious opposition of the British Medical Association and the Pharmaceutical Society, passed through many vicissitudes before it secured the measure of relief which, with its very modest chief organiser, was fêted at the dinner. Viscount Knutsford, to whom Mr. F. A. Hocking (the President of the Association) had yielded the chair for the evening, made use of the opportunity of meeting so many hospital men to talk about hospital matters in general. No doubt Mr. Bridgeman, who is himself Treasurer of the Florence Nightingale Hospital for Women and Chairman of the Supply of Nurses Committee, was quite willing to breathe a little hospital atmosphere, remarking, to the amusement of the company, that in respect of his latest appointment he would at least be armed with the impartiality of ignorance. But those who have studied the methods that brought about his achievement in respect of the spirit duty will be heartened by the knowledge that he also possesses those excellent qualities in dealing with a difficult subject—namely, tenacity and great good humour.

### LORD KNUTSFORD PROPHETIC.

The Chairman of the London paid a well-meant but curiously-worded compliment to hospital secretaries. He said that a chairman is something like a prize carnation—not appearing at its best unless a stick is behind it: the secretary being the stick! He himself once had an ambition to become a secretary; it was at the time when his father was Secretary of State for the Colonies and Mr. Bridgeman was his private secretary. The then youthful Honourable Sydney Holland suggested that he should replace the older man, but the late Viscount Knutsford dryly replied that he preferred Mr. Bridgeman. Having administered one or two sly digs at the College of Nursing and the King's Fund statistics, the Chairman spoke of some of the hospital problems of the future which the younger men present perhaps would have a share in settling. He alluded to the too prevalent inadequate accommodation of nurses, a glaring instance of which is to be found at one of the largest Red Cross hospitals in London (as already reported in The Hospital). Other points were the unsatisfactory geographical distribution of hospitals, the consolidation of smaller medical schools, wider views as to medical women graduates and students, pensions to hospital officers, and paying patients. Those present were asked to take a wide outlook on all these difficult subjects, laying aside unreasonable fears as to a modicum of State or municipal co-operation in the management of our voluntary institutions.

### THE CANADIAN MEDICAL SERVICE.

Hard upon the heels of the Sir Sam Hughes incident, briefly alluded to in these columns a week ago, the controversy between Surgeon-General G. C. Jones and Colonel H. A. Bruce concerning the Canadian Army Medical Service has come to a head. The High Commissioner for Canada has constituted a Board of Inquiry, with instructions to report as quickly as possible upon the criticisms contained in Colonel Bruce's report, and whether they are justified in whole or in part; also upon the recommendations made by Colonel Bruce and whether the board endorses and concurs in them, and if not, in what respects does the board differ in opinion, and its reasons therefor. At the request of Sir George Perley, Sir Alfred Keogh has consented to nominate a British officer as president of the board; and has shown his sense of the importance of the inquiry by nominating his own right-hand man, Surgeon-General Sir William Babtie, K.C.M.G., C.B., V.C. The other members of the board are three Canadian colonels and a lieutenant-colonel; three of these four are officers of the Canadian Medical Service, the other one a combatant officer.

### A NEW HOSPITAL TREASURER AND HIS RECORD.

In the issue of The Hospital of November 4, reference was made to the death of Sir William Vincent, the Treasurer of the Leicester Royal Infirmary. Coincidentally a great effort by Mr. Alfred Corah, J.P., Leicestershire's High Sheriff, in securing upwards of £4,300 in new and increased annual subscriptions was announced. It is not surprising in view of Mr. Corah's long record of usefulness that the Board of Governors of the Leicester Royal Infirmary should have turned to him with a unanimous request to succeed Sir William Vincent as Treasurer to the institution for a period of three years, a request with which he has happily complied. Proved and successful business directors like Mr. Corah, who are willing to devote their time to the work and welfare of the voluntary hospitals, are, in the present crisis, invaluable. The immediate succeeding years of voluntary hospital work are likely to prove of great moment and value to the nation. There will doubtless be a gradual closing down of the temporary military hospitals which have been constructed during the war. This gradual closing down, and the discharge of men from the Army on disbandment, or for other reasons, will inevitably throw great responsibilities upon the voluntary institutions, and it is therefore of the utmost importance that hospital administration shall be highly developed and perfected. The keynote of successful modern businesses is

"organisation." Organisation is highly technical, and, from one point of view, costly, but costliness is but comparative if it brings increased results and improved finances. Coming years will inevitably witness a great development in the business side of hospital administration, and directors of wide outlook like Mr. Alfred Corah, who are at the head of flourishing industries, should prove safe leaders to follow.

### THE ST. GEORGE'S HOSPITAL EXTENSION.

THE new proposals for the extension of St. George's Hospital westwards up to the Hyde Park Corner Tube Station were described in these columns recently (THE HOSPITAL, November 11, 1916; p. 112). It remains only to add on the present occasion that at a special Court of the Governors last week, presided over by Lord Greville, the plans were formally approved. The recommendation of the house committee to the Court that no active steps to carry out the scheme should be taken until after the war was also approved; and it was stated that a rough estimate of the expense of the extensions works out at £74,000.

### THE EXTENSION OF THE SPECIAL BOARD OF MEDICAL APPEAL.

IT appears from certain statements made during the hearing of a case before the City Local Tribunal that the enormous amount of work thrown upon the Board of Medical Appeal has resulted in the overtasking of Sir Frederick Treves and Sir James Mackenzie, both of whom have many other claims upon their time. In consequence of the numbers of cases referred to this special Board, other expert consultants have been added to it, though to the best of our knowledge no public announcement has been issued as to their names. A man in the City area seeking exemption was referred to this Board, where he expected to be examined by one or other of the two original members. Instead he was seen by some other doctor, who passed him for garrison duty abroad, the allocation against which he was appealing. He accordingly conceived that injustice was being done to his claim because he had not been examined by either of the two gentlemen named above, and seems to have thought himself quite entitled to go before them, although he was assured that his examiner was someone of equal eminence and competence. The Tribunal refused to share his view that he had been unfairly treated, in which opinion we concur. Still, it is probable that public confidence in this Appeal Board would be heightened by the knowledge of those who now compose it; and it is advisable that such information should be published, since there can be no possible gain in concealment.

### A MEDICAL APPEAL BOARD FOR MANCHESTER.

AT a meeting of the members of the Manchester Tribunals under the Military Service Acts, held on November 15, it was decided to forward to the War Office a request for a Medical Appeal Board separate from that recently set up in London to deal with disputed medical cases. The contention is that much money and time would be saved were such a Board set up for the Midlands and the North of England; and Manchester claims to be the most suitable centre, both geographically and through its importance as a military centre. It is probably true that a separate Board for the North would be of great value, and it is to be hoped that the War Office will see its way to consent. The only possible objection that can be anticipated may be that only in London are experts to be found whose fiats carry sufficient prestige to be instantly accepted as final. It is true that nowhere outside London can there be found a Sir James Mackenzie, but we hardly think this need weigh the balance down against the other advantages of the suggestion. As regards locality, probably Manchester has few rival claimants to fear, except possibly Leeds. If Scotland wants a separate Appeal Board, Edinburgh would, of course, be the seat of it; but if one Board were set up for Scotland and the North of England one could imagine Newcastle putting forward a claim which would have to be considered.

### A PERTINACIOUS M.P. AND HIS MEMORY.

WE are glad that one of our late politicians, who is really to be lamented because of his pertinacity at question-time, is commemorated by the opening of a pavilion named the "Basil Ward" at the Chesterfield and North Derbyshire Hospital. Given by Mr. C. B. Markham, in memory of Sir Arthur Basil Markham, the late baronet, the new ward-pavilion is intended for wounded soldiers, in the first instance at least. Consisting of weatherproof boards encased within by an asbestos lining, the ward itself is 72 feet long, and the structure measures 106 feet by 22 feet. It comprises a kitchen, a sanitary unit, and two day or living rooms. The donor has also provided the beds and equipment. The total cost is understood to have been £1,500. The new ward raises the accommodation at the disposal of the wounded from thirty to fifty beds. In a speech at the opening, Mr. E. C. Barnes, chairman of the board of management, reminded those present that the connection of the Markham family with the hospital was a long and generous one, and that it was Mr. Markham's father who had first suggested the election of workmen's representatives to the board.

### AN IRISH WIT ON ANNUAL SUBSCRIPTIONS.

A SPEECH of wit and good sense enlivened the recent annual meeting of the National Maternity Hospital, Dublin. As it provides in a very succinct form an argument which is apt to bore subscribers to hospitals when it is laboriously unfolded to them, we quote it in the hope that it may prove useful to hospital administrators in the time of financial trouble. At the meeting in question Lord Justice Malony appealed for annual subscriptions on the amusing ground that they were the life-blood of voluntary hospitals because "people would not die to suit the institution, they died because they could not help it. He often thought, therefore, that it would be better if the present Such-an-one would give something without waiting until he or she became the late So-and-so," Why not? Subscriptions to hospitals have been described by Irishmen as a form of fire insurance, and the sardonic humour of this old joke may serve to remind the public that a yearly "premium" is considered in this world, at all events, a safer policy than one taken out the evening before the fire. That, we believe, is the eternal truth of the matter.

### ULSTER STRIKERS STOP A HOSPITAL BUILDING.

In Belfast of late thirty carpenters, plasterers, stone-cutters, and bricklayers held up by a strike the building of the new wing of the U.V.F. Hospital. Accommodated in old Exhibition Hall, which was converted to hospital purposes, the institution was being added to in response to the demand for beds which was felt, and made consequent upon the great offensive. With a view to adding 150 beds, plans were obtained, and a contract made with a local builder. The work was proceeding when the strike occurred. The strike is understood to have arisen on the refusal of the master-builders to grant a war bonus of 5s. to the men. The importance of the dispute may be judged by a local report that all building in the Belfast area is stopped except in buildings under Government control. Since the war, it appears, the working week of the men affected has fallen from 54 hours to 49½, while they claim that their wages have remained the same, and that their request could not be withheld owing to the rise in prices. Their wages are understood to be £2 a week, and the edge to their grievance seems to lie in their contention that the members of their trade engaged in the shipyards have received the bonus denied to themselves. Meantime the building of the hospital has been interrupted, and one judges that the union controls the bulk, if not all, of the men in the trade.

### THE HEALTH OF MUNITION WORKERS.

Various points connected with the health of munition workers have been dealt with in these columns: their housing, their eyesight, their liability to accidents. Now comes a Memorandum, drafted by the Health of Munition Workers Committee, in which washing facilities and baths are discussed. The Memorandum starts by reminding us that cleanliness is not only valuable in itself, but in the self-respect that it brings, and that bathing facilities in the factories enable a man to enjoy an evening's recreation which would be impossible for him if he had to travel to his home for a bath. On the Continent facilities are said to be much more general than here. Where they prevail, to take an instance, spray baths are provided for foundrymen, at a charge of 3d. for ten tickets, including towel and soap, and seven minutes out of working hours are allowed to each man before stopping-time. The construction of lavatories, made for the hard wear they will receive, and including such points as means for draining the floor, are too much neglected. Very few floors are even sloped, and trouble and expense result from these errors. Plumbing without plugs is illustrated by some ingeniously simple designs for central basins, spray taps, drains flush with the floor, and so forth. The elusive nail-brush can be replaced by a fixed one, which the hand rubs instead of being rubbed by it. Jelly soap, into which fingers can be dipped through a hole, likewise prevents the waste of soap.

### A CURIOUS SUIT AGAINST THE LONDON FEVER HOSPITAL.

A point of considerable interest to nurses and hospital authorities was argued before the County Court Judge at Clerkenwell last week. A nurse, Miss May Knight, of Southampton, aged twenty, asked for an award under the Workmen's Compensation Act against the London Fever Hospital. On September 30, 1915, while disinfecting hairpins belonging to a patient she pricked her index finger. Erysipelas followed, and she lost the use of the finger. In May a doctor advised her that she would be all right if she had a portion of the finger removed, but her counsel now contended that a girl of marriageable age would be most unwise to submit to the operation. In holding that Miss Knight at present was totally incapacitated and entitled to compensation at the rate of 14s. 7d. a week since the time when she left the hospital in July, Judge Roberts said he could not consider what effect the amputation would have from a purely æsthetic point of view. The medical assessor, however, considered that though the loss of two joints would to a certain extent affect the applicant's chance of employment in private practice—in cases where patients were particular, or fidgety and objected to a nurse who had lost a portion of one finger—for hospital work it would not affect her.

### CHRISTMAS AT THE CARDIFF MILITARY HOSPITALS.

The ladies of Cardiff have taken the leading part in a movement which aims at making Christmas a happy season for the 3,000 wounded soldiers who are accommodated at Cardiff and Barry hospitals. Mainly through the influence of Lady Tatem, a sum of over £1,000 has been obtained—without recourse to a Flag Day either—and this and more will be expended on the Yuletide entertainment of the military patients at the various hospitals. Each man is to receive an old-fashioned Christmas dinner, a cigarette-case full of cigarettes, and a walking-stick.

### SANATORIUM FOR TUBERCULOUS LONDON CHILDREN.

According to a statement in the *Times*, there are a thousand children in the London elementary schools suffering from tuberculosis, though it is not clear whether this means tuberculosis of the lungs alone, or whether glandular, joint, visceral, and skeletal tuberculosis is included also. Open-air daily schools provide accommodation for one hundred and ninety of these children, and it is proposed to open two more in the neighbourhood of London. An even more satisfactory way of dealing with these cases has been devised at the well-known sanatorium directed by Dr. Jane Walker at Nayland, in Suffolk, where provision has been made for one hundred children to live a completely healthy outdoor life for six months at least apiece. Early this year extra buildings were opened at Nayland for the accommodation of one hundred children up to the age of fifteen. Most of the cases are sent by the London County Council, others by the Essex Council, by Boards of Guardians, and by private agencies. They are paid for at the rate of 23s. a week for a minimum period of six months. The buildings consist of an administrative central block, separating two pavilions, one for each sex. Each pavilion has four wards, with six or seven beds in each; one side of each ward is entirely open. Systematic instruction is carried on; though, owing to the physical condition of the children, it has to be limited to three hours a day, equally divided between morning and afternoon. The results obtained are stated to be highly satisfactory from the medical point of view, as might be expected.

### A POINT FOR SANATORIUM ARCHITECTS.

A curious point, well worthy of the attention of those who select sanatorium sites or plan sanatorium buildings, was raised at the last Court of Governors of the Brompton Hospital for Consumption and Diseases of the Chest, held on November 17. It seems that on certain land adjacent to the sanatorium at Frimley, belonging to this hospital, there exists a grove of pines which shelters the institution very markedly from the prevailing gales. The demolition of these pines was threatened, and this prospect was thought to be so disadvantageous to the patients at the sanatorium that, in order to avert it, the purchase of the land on which the pines stand appeared advisable. The moral of this incident is plain enough not to need underlining, and can be commended to the attention of sanatorium managers, committees, and architects. The committee of management of the Brompton Hospital reported to the Court, it may be added, that although the general response to the late appeal for funds has been disappointing, sufficient has been raised for the purchase of this tract of land: from which it is perhaps to be inferred that the question of the pine-trees is regarded as the most urgent and important of the needs of this institution at the present time.

### THE SHAFTESBURY GUARDIANS AND THEIR MEDICAL OFFICER.

After a somewhat prolonged controversy, the Shaftesbury Guardians have arrived at a settlement of the dispute they have been conducting with the Local Government Board concerning the salary paid to their medical officer, though it is interesting to note that in order to arrive at this settlement the board had to throw over their own chairman and vice-chairman. Briefly, the issue was whether the salary of the medical officer should be reduced. The Guardians wished to cut it down, on the (alleged) ground that less work was being required of this officer than was formerly the case. The Local Government Board had to point out to the Guardians that such a reduction cannot legally be carried out without the consent of the former body (or of the medical officer in question). And the Local Government Board further, after hearing the pros and cons of the case, refused to give the necessary assent. A sub-committee of the Guardians, however, tendered a bellicose report, arrived at by a majority of three to one: the chairman and vice-chairman of the Guardians were two of the three fire-eaters of the majority. This report was a vindication of the proposal to reduce the salary, and invited the Guardians to fight the Local Government Board. A minority report was presented by the dissenting member of the sub-committee, and was adopted by the Guardians at their meeting on November 9 by a large majority. The result is that the medical officer will continue to receive his former salary, and that the Shaftesbury Guardians have bowed to the authority of the Local Government Board: in both actions we conceive that they have taken a wise decision.

### A NEW INSTITUTION FOR THE AGED AND FOR WAR INVALIDS AT KIRKCALDY.

A legacy of nearly £30,000 was announced at the last meeting of the Kirkcaldy Town Council, when it was reported that Mr. John Hunter, of St. Brycedale House, Kirkcaldy, who died a fortnight ago, had left practically his entire estate, estimated to produce over £2,000 a year, in trust for the conversion of St. Brycedale House into a hospital for the aged and incurable, and for providing allowances for the wounded sailors and soldiers of the borough, any surplus income after meeting these trusts to be distributed among the poor of the town.

### A REMARKABLE INQUEST CASE.

A week or two ago an inquest was held in London which resulted in the vindication of a medical man from certain serious charges. The proceedings point three useful morals, two of them very old indeed and the other not new, but well worth restating all the same. The subject of the inquest was a woman whose death was clearly due to an illegal operation. She made three separate "dying declarations" in due form, which were therefore admissible as evidence. There were minor variations in these declarations, but substantially they agreed in attributing to a certain practitioner two (unsuccessful) attempts to procure abortion upon her, followed by a (successful and fatal) attempt at the hands of a woman. The implicated practitioner, fortunately for him, had kept his books in good order; and was able by means of them to show that the woman had seen him only once, on which occasion she gave a false name; had paid him a fee of five shillings; and had received from him a prescription for a quite normal tonic medicine. According to his evidence she had requested him to undertake the illegal operation, which request he had refused. The three lessons of this case are, first, the necessity of punctual and precise bookkeeping; secondly, the importance of joining the Medical Defence Union, whose solicitor attended to represent the doctor; and lastly, the limited credence to be attached to dying declarations. Presumably, the fear of speedy dissolution was not sufficient to deter this woman from bringing a false charge against the doctor; and the only plausible motive for this extraordinary act would seem to be a desire for revenge upon the doctor for refusing her the performance of the operation she desired.

### THE WAR OPENS THE WAY FOR MEDICAL WOMEN.

An unprecedented step, as a politician would say, has been taken by the Leeds Guardians, who have at last decided to appoint a woman medical officer and not to advertise the post as open to either sex. Before the war this suggestion gained few supporters in Leeds, but now a jog from the Local Government Board inspector, aided by circumstances, has produced a change of policy. It is true that the inspector recommended the appointment of two women doctors, and backed his proposal by pointing out that some 800 patients have been in the charge of two medical officers only. The *Yorkshire Observer* remarks that the Leeds public hospitals already possess two women medical officers in Dr. A. Umansky, at Killingbeck, and Dr. Agnes Andrew, at Seacroft, while by a recent decision of the Sanitary Committee a woman doctor is being appointed as assistant in connection with the Leeds infant-welfare scheme. So, as generally turns out on examination, an unprecedented step is not necessarily such a very dreadful thing after all.

### PARLIAMENTARY REPRESENTATION FOR PHARMACY.

Mr. W. S. Glyn-Jones, M.P. for Stepney, has announced that owing to ill-health he will not seek re-election to Parliament at the next general election. Mr. Glyn-Jones has been for some years Parliamentary Secretary to the Pharmaceutical Society of Great Britain, and has ably looked after the interests of pharmacy in matters affecting the profession—notably in the passage of the "1908 Poisons and Pharmacy Act" which, amongst other things, stipulates that in every chemist's shop the certificate of the pharmacist in charge thereof must be exhibited in a prominent place. The Pharmaceutical Society has decided to permit their Secretary and Registrar, Mr. W. J. U. Woolcock, to become a candidate for a seat in Parliament, and he has been nominated as prospective candidate for Central Hackney. Mr. Woolcock was for some years a hospital pharmacist; he was chief assistant dispenser at University College Hospital, during which period he qualified as barrister-at-law. With the many problems frequently arising regarding the dispensing under the National Health Insurance Act and other matters concerning pharmacy, a representative in Parliament should be advantageous to pharmacists. The many institutional workers who know Mr. Woolcock, will wish him success in the political world.

### A WEST-COUNTRY TRAINING SCHOOL'S RECORD.

Fitting emphasis was laid on the importance of child-welfare and maternity work by Sir Henry Lopes in presiding at Plymouth last week over the annual meeting of the Devon and Cornwall Training School and the Three Towns Nursing Association. Sir Henry paid a tribute to the great value of the institutions, both in general nursing and on the maternity side, but added that the time had come when voluntary effort should be helped by public funds or money from the rates. Funds are, of course, already available through the medium of the Local Government Board, and, though the West Country has been slow in putting its house in order so as to claim the financial assistance which the Government offers, two towns—Exeter and Plymouth—at least have made efforts to keep abreast of the times in this vitally important matter. Dr. Clay told the meeting something of the work which the Three Towns Nursing Association accomplished last year. Nearly 52,000 visits were paid by the staff, and the tuberculosis dispensary proved a valuable adjunct. Infant-welfare work has lately been taken up, and the last attendance at the institution was 110 mothers and 86 children. This branch of the work has so developed that it is necessary to add to the premises a room to hold 150 chairs, a waiting-room, and a doctor's room. This extension will cost £500.

### AN OBJECTIONABLE CIRCULAR.

A marked affront, in our opinion, has been offered to members of the medical profession by the publishers of the annual "Medical Who's Who" in a circular which has been sent to some, at least, of the doctors whose names appear in it. The circular dwells upon the increased cost of paper and of printing, and the consequent necessity of abbreviating "many of the lengthy biographies" supplied to the publishers in response to their own requests in previous years. No one has any right to cavil at this procedure, especially in view of the sound argument upon which it is based; but what the doctors have a right to protest against, as one of them has already done in drawing our attention to the circular, is the succeeding paragraph of the circular, which sets out that in the case of subscribers the editor of the "Medical Who's Who" will endeavour to include as much information as possible. Most doctors, it may be imagined, to whom this extraordinarily tactless and clumsy hint has been distributed will feel inclined to request the omission of their biographies altogether from a publication conducted upon such equivocal lines. In justice to the original publishers of this useful publication, it is only fair to say that hitherto it has been managed on the most completely unobjectionable principles.

### A DEPLORABLE CONFLICT OF MEDICAL EVIDENCE.

At the Newcastle County Court was recently heard a compensation suit wherein a most deplorable conflict of medical evidence was manifested. Incidentally, the Judge might have profited by the services of a medical assessor of high standing, which are always available to assist the Courts in such cases. The case concerned a stereotyper in a printing firm, who died in May this year. The contention of the widow (the appellant) was that death was due to lead poisoning from the fumes of molten metal near which the deceased did his work; the contention of the defence was that death was due to tuberculosis of the lungs, and had nothing to do with lead poisoning. In support of the appellant's case Sir Thomas Oliver and the patient's panel doctor gave evidence. The latter had to admit that he had attended the man for some months for bronchitis and had not suspected lead poisoning until a few days before death. He is also reported to have said that at the *post-mortem* examination he found " traces of tubercular disease of the lungs of long standing "; a statement which on cross-examination he was induced to modify into " cavities in the lung full of pus," indicative of " an advanced stage of the disease." The other medical witnesses pooh-poohed the diagnosis of lead poisoning; and the Judge decided against Sir T. Oliver's contention. As far as can be judged from the report, his decision was eminently sensible; conflicts of expert evidence of this kind are simply deplorable, and go far to strengthen the contention of some authorities that litigants should not be allowed to produce and pay their own selected medical witnesses in this type of case.

### THE FEAR OF HOSPITALS.

Even now people resent the establishment of hospitals in their midst, and the Urban District Council of Ware has lodged with the County Authority a protest against the proposed establishment of a hospital for tuberculosis as likely to affect injuriously the prosperity of the town, as being a source of risk of danger to the health of the inhabitants, as a probable cause of deterioration in the value of the property in the town and neighbourhood, and as being provided on a site in an unsuitable position for the purpose. Local inhabitants have also petitioned against the scheme, but such protests are now too late. The Hertfordshire County Council has proceeded too far with the scheme to withdraw, and replies to the protests by saying the people will not find the institution in any way injurious to the health of the inhabitants. The Local Government Board has now sanctioned the plans for the adaptation of the premises, known as " Canons," as a tuberculosis hospital, at a cost of £800.

### HOSPITALS AND THE PRICE OF NEWSPAPERS.

Our voluntary hospitals and charities generally owe so much to the publicity given to their needs and work in the columns of the newspapers, without whose aid appeal-advertisements would be much restricted in value, that they and their supporters may well give an intelligent thought to the condition of newspaper men and newsvendors. The reduction in the size of most newspapers and the common use of cheaper paper were the first signs that the public had of the difficulties created by the war for journalists and newsmen. A still more significant move is the raising of the price of London's most pretentious journal, a move which may or may not be anticipating the plans of other publications. The point for hospital men in particular, however, is that while such changes may be necessary they indicate the position of newspaper staffs and newsmen, whose incomes and needs have *not* changed and are not changing with the rise in prices and the difficulties of their trade. We therefore commend to all alert enough to grasp what the hospitals owe to journalists and newsvendors the plea of the Newsvendors' Benevolent and Provident Institution, which is badly in need of funds, and which (hospital secretaries please note) is abandoning its usual yearly festival. Subscriptions may be sent to Lord Burnham at the institution's offices, 15, 16 Farringdon Street. A fraction of what newspapers have raised for charities during the war alone, if sent to the Newsvendors' Institution, would place it on its legs for a long time to come.

### THIS WEEK'S DRUG MARKET.

The improvement in business which was noticeable a week ago has not been maintained, and the market is again rather quiet. The demand for quinine has apparently been satisfied, and no business of importance has taken place during the week; the price, however, remains unchanged for the time being. The price of camphor is well maintained, and as English refiners are full up with orders, there would appear to be no prospect of any reduction in the near future; on the contrary, a further advance in price would not come as a surprise. Colchicum root is scarce, with an upward tendency in price. Menthol is still in good demand, and the price is again rather higher. Orris root is extremely scarce and very dear. Stocks of juniper oil are steadily dwindling, and as they cannot be replenished, the scarcity will soon be very pronounced. Bergamot oil has a lower price tendency. Pilocarpine and sparteine are both very scarce. There is still a shortage of phenacetin. Aspirin, salicylic acid, and sodium salicylate are all rather cheaper; and in view of the increased output the decline in values may continue. Epsom salts and castor oil are tending upwards in price, the tendency being very pronounced in the case of the latter drug. At the recent public sale of drugs in Mincing Lane there was a good demand for honey, which sold at rather higher rates. Senna leaves also sold well; the price of sarsaparilla was well maintained, and there was a better demand for cardamoms.

### TO OUR READERS.

**Contributions are specially invited from any of our readers to these columns. They should deal with topical subjects and news. They must be authenticated for the information of the Editor only. The minimum payment if published is 5s. There is no hard-and-fast rule as to space, but notes of about twenty lines in length are preferred.**

